# Intratumoral microbiome of adenoid cystic carcinomas and comparison with other head and neck cancers

**DOI:** 10.1038/s41598-024-65939-9

**Published:** 2024-07-15

**Authors:** Tatiana V. Karpinets, Yoshitsugu Mitani, Chia-Chi Chang, Xiaogang Wu, Xingzhi Song, Ivonne I. Flores, Lauren K. McDaniel, Yasmine M. Hoballah, Fabiana J. Veguilla, Renata Ferrarotto, Lauren E. Colbert, Nadim J. Ajami, Robert R. Jenq, Jianhua Zhang, Andrew P. Futreal, Adel K. El-Naggar

**Affiliations:** 1 Department of Genomic Medicine, The University of Texas MD Anderson Cancer Center, Houston, TX USA; 2 Department of Pathology, The University of Texas MD Anderson Cancer Center, Houston, TX USA; 3 Department of Thoracic/Head and Neck Medical Oncology, The University of Texas MD Anderson Cancer Center, Houston, TX USA; 4 Department of Radiation Oncology, The University of Texas MD Anderson Cancer Center, Houston, TX USA

**Keywords:** Oral, Bacterial, Mucus layer, Tumors, *Bacteroides thetaiotaomicron*, Cancer, Computational biology and bioinformatics, Microbiology, Molecular biology

## Abstract

Adenoid cystic carcinoma (ACC) is a rare, usually slow-growing yet aggressive head and neck malignancy. Despite its clinical significance, our understanding of the cellular evolution and microenvironment in ACC remains limited. We investigated the intratumoral microbiomes of 50 ACC tumor tissues and 33 adjacent normal tissues using 16S rRNA gene sequencing. This allowed us to characterize the bacterial communities within the ACC and explore potential associations between the bacterial community structure, patient clinical characteristics, and tumor molecular features obtained through RNA sequencing. The bacterial composition in the ACC was significantly different from that in adjacent normal salivary tissue, and the ACC exhibited diverse levels of species richness. We identified two main microbial subtypes within the ACC: oral-like and gut-like. Oral-like microbiomes, characterized by increased diversity and abundance of *Neisseria, Leptotrichia, Actinomyces, Streptococcus, Rothia,* and *Veillonella* (commonly found in healthy oral cavities), were associated with a less aggressive ACC-II molecular subtype and improved patient outcomes. Notably, we identified the same oral genera in oral cancer and head and neck squamous cell carcinomas. In both cancers, they were part of shared oral communities associated with a more diverse microbiome, less aggressive tumor phenotype, and better survival that reveal the genera as potential pancancer biomarkers for favorable microbiomes in ACC and other head and neck cancers. Conversely, gut-like intratumoral microbiomes, which feature low diversity and colonization by gut mucus layer-degrading species, such as *Bacteroides, Akkermansia, Blautia, Bifidobacterium,* and *Enterococcus*, were associated with poorer outcomes. Elevated levels of *Bacteroides thetaiotaomicron* were independently associated with significantly worse survival and positively correlated with tumor cell biosynthesis of glycan-based cell membrane components.

## Introduction

Adenoid cystic carcinoma (ACC) is a malignancy of major and minor salivary glands and less commonly of other organs^1^. It is a slow-growing aggressive tumor characterized by frequent local recurrence and delayed distant metastasis^2^. The ACC is subdivided into 3 histological groups (tubular, cribriform, and solid)^3^ and into 2 molecular subtypes, ACC-I and ACC-II. The former is characterized by activation of MYC and MYC target genes, enrichment of NOTCH-activating mutations, and significantly worse overall patient survival than ACC-II^4^. There are few data on the factors underlying the development and progression of this disease^2,5^. A recent small study of saliva from 13 ACC patients and 10 healthy controls reported significant differences in the taxonomic structure of the salivary microbiome between these 2 groups, with greater abundances of *Rothia* and *Streptococcus* in the saliva of ACC patients^6^. These findings highlight the difference between the intratumoral microbiome and normal tissue and suggest a role for the salivary microbiome as a potential biomarker of ACC.

Although the intratumoral microbiome has not been studied in the ACC, numerous studies of bacterial communities in solid cancers, including colorectal^7^, oral^8^, pancreatic^9,10^, and gynecological cancer^11^, revealed differences in bacterial diversity and taxonomic composition between tumors and normal tissues. While challenges in definitively determining the abundance, roles, and consequences of intracellular bacteria in cancer remain^12^, emerging evidence strongly suggests their potential for the diagnosis, prognosis, and treatment of various tumors^13–15^. Both positive and negative associations of intratumoral bacteria with tumor development, progression, patient survival, and response to therapies have been reported with different underlying molecular mechanisms^16^^,^^17^. Studies have linked certain microorganisms to the prognosis and treatment of cancer including pancreatic^18^, colorectal^7^, and others^19^.

An important function of the salivary gland is the secretion of saliva, which contains antimicrobial factors that impact microorganisms^20,21^ and may play a role in the transformation of salivary epithelia and other cells during the course of ACC development, progression, and treatment. Salivary gland-derived mucins are important components that mediate the interactions of microorganisms with epithelial cells and their environment^22^. Mucins are glycoproteins produced by epithelial cells. They are comprised of a protein and a glycan moiety with recognition motifs and binding sites for microbes^23^. The interaction of microbes with mucins plays a central role in maintaining oral health. However, how ACC development impacts microbial diversity, taxonomic organization, and interactions between microbes and salivary gland components is unclear.

In this study, we analyzed the taxonomic structure and diversity of the intratumoural microbiomes of ACC tumors and adjacent nontumoural tissues using 16S RNA gene sequencing, explored the associations of the microbiomes with clinical characteristics and molecular features inferred by RNA sequencing, and compared the intratumoural microbiomes of ACC patients with those of patients with oral and head and neck cancers.

## Methods

### Study design

Fresh-frozen tumors of 50 ACC patients were available for 16S rRNA gene sequencing (Supplementary Fig. S1). Histologically nontumoral adjacent tissue was available for only 33 of these patients. We sequenced a total of 88 samples (Supplementary Fig. S2). This included 33 paired tumor-normal samples (66 samples), 17 additional tumor samples without paired normals, and 5 duplicate samples to assess potential batch effects. The IDs of all sequenced samples, along with other relevant clinical information for the patients, are provided in the supplementary table (Supplementary Table S1) and are available for download as raw sequencing reads from the NCBI Sequence Read Archive.

### DNA extraction and 16S rRNA gene sequencing

Snap-frozen human tumor and normal tissue specimens were processed and analyzed at the MD Anderson Cancer Center Microbiome Core Facility. Microbial genomic DNA was extracted using a DNeasy Powersoil Pro DNA Kit (Cat No. 47014, QIAGEN) following the manufacturer's instructions. The extraction process involved adding 100 mg to 200 mg of homogenized adenoid cystic carcinoma (ACC) or corresponding normal tissue to a bead beating tube for efficient homogenization and lysis using mechanical and chemical methods. Subsequently, the lysed cells were treated with Inhibitor Removal Technology (IRT) solution to remove the inhibitors. The total genomic DNA was then captured on a silica membrane in a spin-column format, followed by washing and elution steps. The Earth Microbiome Project^24^ method was used to construct amplicon libraries of the V4 hypervariable region of the 16S rRNA gene. PCR amplification of microbial DNA was carried out using the 515F and 806R primer sets, which contained sequencing-ready barcodes and adapter sequences. The quality and quantity of the barcoded amplicons were assessed using an Agilent 4200 TapeStation system (Agilent) and a Qubit fluorometer (Thermo Fisher Scientific). The amplicons were pooled in equimolar ratios. The pooled libraries were quantified using a Qubit fluorometer, and their molarity was calculated based on the amplicon size. Sequencing was performed on the Illumina MiSeq platform (Illumina) using the 2 × 250 bp paired-end protocol, which resulted in paired-end reads with near-complete overlap. The custom sequencing primers used were as follows: Read1 sequencing primer: 5′-TATGGTAATTGTGTGYCAGCMGCCGCGGTAA-3′; Read2 sequencing primer: 5′-AGTCAGCCAGCCGGACTACNVGGGTWTCTAAT-3′; and index sequencing primer: AATGATACGGCGACCACCGAGATCTACACGCT. The sequencing data from the paired-end reads were demultiplexed using QIIME^25^. The paired-end reads were merged, followed by dereplication and length filtering using VSEARCH v7^26^. Denoising and chimera calling were performed using the unoise3 command^27^. Bacterial taxonomies were assigned using the SILVA database version 138 (https://www.arb-silva.de/)^28^.

### Statistical analyses

Bacterial diversity was calculated at the level of predicted OTUs and at the genus level in terms of different diversity measures using the R library ‘microbiome’^29^. The presence of differentially abundant taxa between 33 paired tumor tissues and adjacent normal tissues was determined by 2 different tools, MaAsLin2^30^ and LeFSe (logarithmic discriminant analysis effect size)^31^, using default parameters. In the case of LeFSe, the logarithmic discriminant analysis (LDA) score threshold was set to 1.5 to identify additional differentially expressed taxa.

Supervised hierarchical clustering of the most common OTUs identified in ACCs was implemented by the open source clustering software Cluster 3^32^ with default parameters using centroid linkage as the clustering method. The samples were ordered by the abundance of *B. thetaiotaomicron* and by the sum of the 3 species abundances, namely, *Granulicatella adiacens*, unclassified *Leptotrichia*, and *Rothia mucilaginosa,* for the *B.thetaiotaomicron*-negative samples*.*

Permutational multivariate analysis of variance (PERMANOVA) and nonparametric multivariate analysis of variance (MANOVA)^33^ were applied to 33 normal and tumor pairs to test whether the groups had distinct taxonomic compositions and to 50 ACC tumor samples to test the association of the proposed grouping of the samples with taxonomic composition. The analysis was implemented using the R libraries “vegan”^34^ (https://rdrr.io/cran/vegan/, R package version 2.6-4) and the functions adonis2() and permutest(). The default parameters were used if they were not specified differently in the text.

Associations with survival were explored for the most common and abundant OTUs identified in 50 ACC tumors using the Cox proportional hazards model: coxph() function in the R library "survival"^35^ and the R library "survminer"^36^. Namely, we aggregated the produced OTU table at the species level and selected those OTUs that are most common in ACC tumors (found in more than 20 samples out of 50). The significance of associations was evaluated by the log-rank test, likelihood test, and Wald test. Bonferroni correction^37^ was used to adjust the p values.

Gene set enrichment analysis (GSEA) was implemented for 44 samples with available RNA-seq data using the R package “fgsea” (fast preranked GSEA)^38^ downloaded from https://github.com/ctlab/fgsea. Ranking of genes for the analysis was performed using Spearman correlation coefficients calculated between *B theta* abundances and the gene expression profile for each gene across 44 samples. The gene sets representing KEGG pathways (https://www.gsea-msigdb.org/gsea/msigdb/human/genesets.jsp?collection=CP:KEGG)^39^ and Gene Ontology Biological processes (https://www.gsea-msigdb.org/gsea/msigdb/human/genesets.jsp?collection=GO:BP)^40^ were downloaded from the Molecular Signature Database v2022.1^41^.

Comparison of the ACC bacterial community structure with that of oral cancer was performed using a recently published cohort of 33 oral cancer patients with tumors analyzed by 16S RNA gene sequencing^8^. The OTU table was aggregated to the *genus* level in each of the 2 cohorts, referred to as Oral and ACC. The most common genera (found in 75% of samples) were selected for unsupervised hierarchical clustering implemented separately for each cohort by open source clustering software Cluster 3^32^ with default parameters using centroid linkage as the clustering method.

Comparison of the ACC with head and neck squamous cell carcinoma was implemented using predicted bacterial genera in the TCGA-HNSC cohort. The dataset is described by Gihawi et al.^42^ and was available for download as a supplementary table. Briefly, the OTU table was created by refiltering the unmapped reads by aligning them to the human CHM13 reference genome and by matching the refiltered reads with a curated Kraken database^43^. We selected only primary tumors that were fresh frozen and analyzed by WGS using the Illumina HiSeq platform for the comparison. The OTU table of the obtained 170 samples was further filtered by including the 50 topmost common bacterial species to reduce the scarcity of the table for unsupervised hierarchical clustering, which was implemented as described above. Clinical information for the patients was downloaded from the TCGA using the R library ‘TCGAbiolinks’^44^.

### Ethical approval

The study was conducted in compliance with the Declaration of Helsinki and approved by the University of Texas MD Anderson Cancer Center (MDACC) institutional review board (IRB). Samples were obtained by informed consent of the participants. In case of deceased patients, the IRB (the University of Texas MD Anderson Cancer Center institutional review board) approved the waiver of informed consent.

## Results

### Clinical characteristics of the cohorts

Fresh frozen primary tumors from 50 patients with ACC, stage 1–4, and no prior therapy at the time of surgery were subjected to 16S RNA gene sequencing to characterize the intratumoral bacterial composition, diversity, structure, and association with clinicopathological factors (Supplementary Fig. S1). Adjacent nontumoral tissue from thirty-three patients was also sequenced for comparative analysis of microbial communities with those in the tumor. The tumors of 44 patients (88%) were analyzed using RNA-seq in our previous study^4^. A classification of these tumors into 2 molecular subtypes, referred as ACC-I and ACC-II, was available for correlative analysis with the intratumoral microbiome. Patients with both molecular subtypes (45% ACC-I and 55% ACC-II) were included in the study cohort (Table 1, Supplementary Table). The primary sites of ACC in the cohort were glands characterized by the following locations: maxillary sinus, base tongue, palate, parotid, trachea, sublingual, lacrimal, and submandibular. The sites are close to the oral, throat, nasal, and eye ocular surface body sites characterized by the Human Microbiome Project. Most of the patients were males (66%), had perineural invasion (PNI) (84%), and stage 3–4 tumors at diagnosis (70%) with solid or cribriform histology (84%). All the patients underwent primary tumor resection, and most of them were treated with postoperative RT (88%) and/or chemotherapy (38%).

**Table 1:** Clinicopathological characteristics of the ACC cohort.

Characteristics	Number of tumors	%
Age (years)	50	
≤ 50	23	46
> 50	27	54
Histological subtype	47	
Cribriform	19	40
Solid	23	49
Tubular	5	11
Tumor stage	45	
1	3	7
2	7	16
3	14	31
4	21	47
M stage	46	
0	37	80
1	9	20
Nodal status, cN	45	95
N0	34	76
N1	5	11
N2	6	13
Sex	50	50
Male	33	66
Female	17	34
Chemotherapy	50	50
Yes	19	38
No	31	62
Adjuvant RT	49	
Yes	44	90
No	5	10
PNI	46	
Neg	4	9
Pos	42	91
ACC type (RNA-seq)	44	
ACC I	20	45
ACC II	24	55
Primary site	45	
Minor	35	78
Major	10	22

### The taxonomic structure of the bacterial community in ACC tumors and adjacent normal tissue is associated with bacterial richness

**Figure 1:**
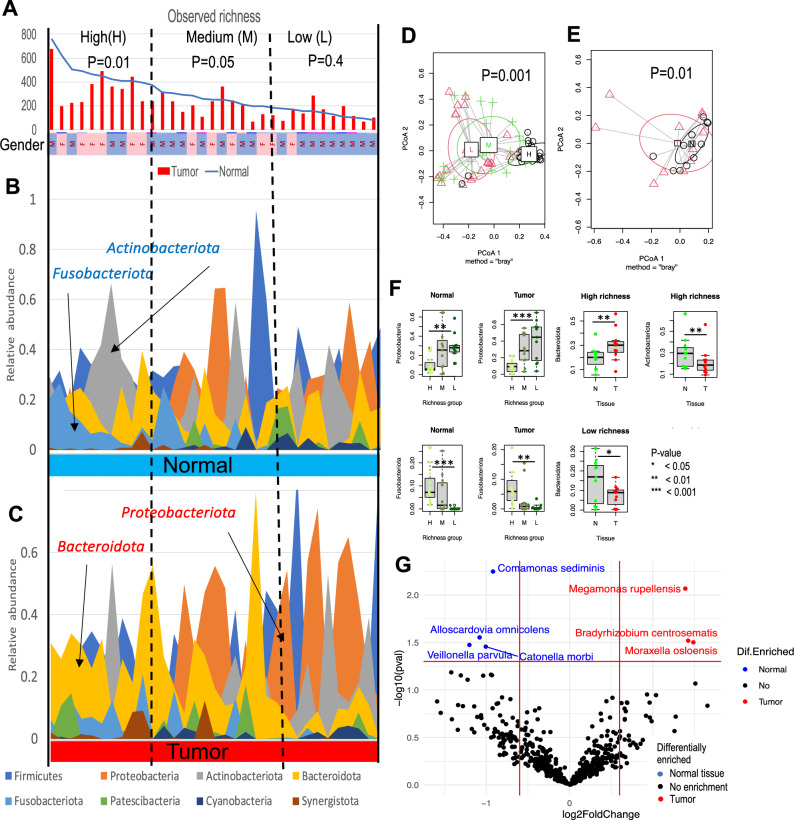
Associated changes in the richness and taxonomic structure of bacterial communities in tumor and matched normal tissues. (**A**) A significant difference in the number of identified spp. (richness) in tumors vs normal tissue was mostly observed in patients with a more diverse microbiome in normal tissue. The samples were sorted by richness and then divided into 3 equal richness groups (see dashed lines): low (L), medium (M), and high (H) number of species in the normal samples. A paired two-sided t test was used to evaluate whether tumor tissue was significantly different from normal tissue in each group. The p values produced from the test are shown in the figure. The sex of the patient is labeled as a bar on the Y axis with a rose (female) or blue (male) color. (**B**) Permutational multivariate analysis of variance of paired tumor-normal tissue samples in terms of bacterial species abundances revealed a significant association between the bacterial community structure and microbiome richness. Centroids of samples for each richness group are labeled H (High richness), M (Medium), and L (Low). (**C**) Permutational multivariate analysis of variance of paired tumor-normal samples from patients in the high-richness group revealed significant differences in the community structure between tumor and normal tissues. Centroids for tumor (red triangles) and normal (black circles) samples are labeled T and N, respectively. (**D**,**E**) Relative abundance of the most abundant phyla in normal and tumor tissues sorted by species richness in normal tissue. The phyla that are significantly more abundant in normal or tumor tissues are labeled with blue and red arrows, respectively. An increased abundance of *Proteobacteriota* and a decreased abundance of *Actinobacteriota* and *Fusobacteriota* were observed in tumors with low species richness. (**F**) Significant difference in the relative abundances of *Bacteroidota, Fusobacteriota* and *Actinobacteriota* between tumor and normal tissue from different richness groups. (**G**) Volcano plot of differentially abundant species identified by MaAsLin in normal and tumor tissues.

16S RNA sequencing of 50 ACC tumors revealed a median number of 200 putative bacterial species per sample, ranging from 52 to 673 OTUs. Further paired comparisons of 33 tumor tissues and normal adjacent tissues in terms of diversity metrics revealed significantly increased species richness (not adjusted two-tailed paired t test P value = 0.005) and related characteristics, Fisher’s diversity and the Chao1 index (P = 0.005), in normal samples. No significant differences (P > 0.05) in other indices of diversity were found between normal and tumor tissues. Further analysis revealed significant pairwise correlations (R = 0.73, P < 1E−5) of the species richness between tumors and paired normal tissues (Supplementary Fig. S2). A significant decrease in the number of species within the tumor was observed only in patients with a rich microbiome; no association was found when the tissue had low richness (Fig. 1A, Supplementary Fig. S3). There was also a trend (Fisher test P = 0.09) for females rather than males to have richer microbiomes in both tumor and normal tissues.

The taxonomic structure of the bacterial community in normal and tumor samples also depended on species richness (Fig. 1B,C respectively). By applying permutational multivariate analysis of variance to all 33 paired tumor-normal samples, we observed significant differences in taxonomic composition when the paired samples were divided into 3 equal groups according to normal tissue richness: high (H), medium (M), and low (L) (Fig. 1D). The community structure of the H group differed from that of the M group and, especially, the L group at both the OTU level (Fig. 1D) and the *Phylum* level (Supplementary Fig. S4B). No difference was found between tumor and normal tissues when all the samples were analyzed together (Supplementary Fig. S4A). However, when examining each group separately, a significant difference in taxonomic structure between tumor and normal tissue was observed in the H-rich group of samples (Fig. 1E and Supplementary Fig. S4C) but not in the M or L group (Supplementary Fig. S4D). Due to inconsistent changes between the groups, more significant differences in the relative abundances of *Phyla* were observed if samples in the L and H richness groups were considered separately (Fig. 1F, Supplementary Figs. S5, S6). Normal and tumor tissues with low versus high species richness showed a significant increase in the abundance of *Proteobacteriota* (P = 0.001 and P = 0.0007) and a significant decrease in *Fusobacteriota* (P = 0.0003 and P = 0.002) (Fig. 1F). Tumors versus normals in H group (with high number of OTUs) showed increase of oral *Bacteroidota* and decrease of oral *Actinobacteriota*, and tumors versus normals in L group (low number of OTUs) showed decreased abundance of *Bacteroidota* (Fig. 1F) and increased abundance of *Proteobacteriota* (Supplementary Fig. S6).

Associations between taxonomic structure and richness were also confirmed at the *order* level (Supplementary Fig. S7) and at the OTU level (Supplementary Fig. S8, Fig. 1G, Supplementary Fig. S9). Differentially abundant OTUs between normal and tumor tissues were found by comparison of 33 paired ACC tumor-normal tissues using MaAsLin2 (Fig. 1G) and LeFSe (Supplementary Figs. S8, S9, S10). Four putative species, including the typical inhabitants of *the* human oral cavity *Veillonella parvula *^45^*, Catonella morbi *^46^ and *Alloscardovia omnicolens*^47^, were abundant in normal tissue (Fig. 1G) and, specifically, in samples with a total number of species above the median value (Supplementary Fig. S9). The opposite relationship with species richness was found for 2 putative species differentially abundant in tumors (Supplementary Fig. S10): the gut bacterium *Megamonas rupellensis*^48^ and *Bradyrhizobium centrosematis*, which are known contaminants of purified and municipal water systems^49^. Both species were abundant in tumor tissue with low species richness. Moreover, *Moraxella osloensis, which is* implicated in ocular membrane inflammation^50^, was more abundant in tumor tissue but was not associated with species richness.

### Association of bacterial composition and abundances with overall patient survival

To identify clinical factors and specific intratumoral species that might be associated with survival outcome, we analyzed tumor tissue from 50 patients (Supplementary Fig. S1). Analysis of clinical factors using the Cox proportional hazards model identified only one confounder, the ACC molecular subtype (ACC-I versus ACC-II), which was significantly associated with survival after multiple testing correction (log-rank Padj = 0.003) (Supplementary Table S2). Among the diversity characteristics, only the diversity inverse Simpson, diversity coverage, and Gini inequality indices showed statistical significance (log-rank Padj < 0.05), although they were not independent predictors of survival probability from the ACC molecular subtype (Supplementary Table S2).

**Figure 2:**
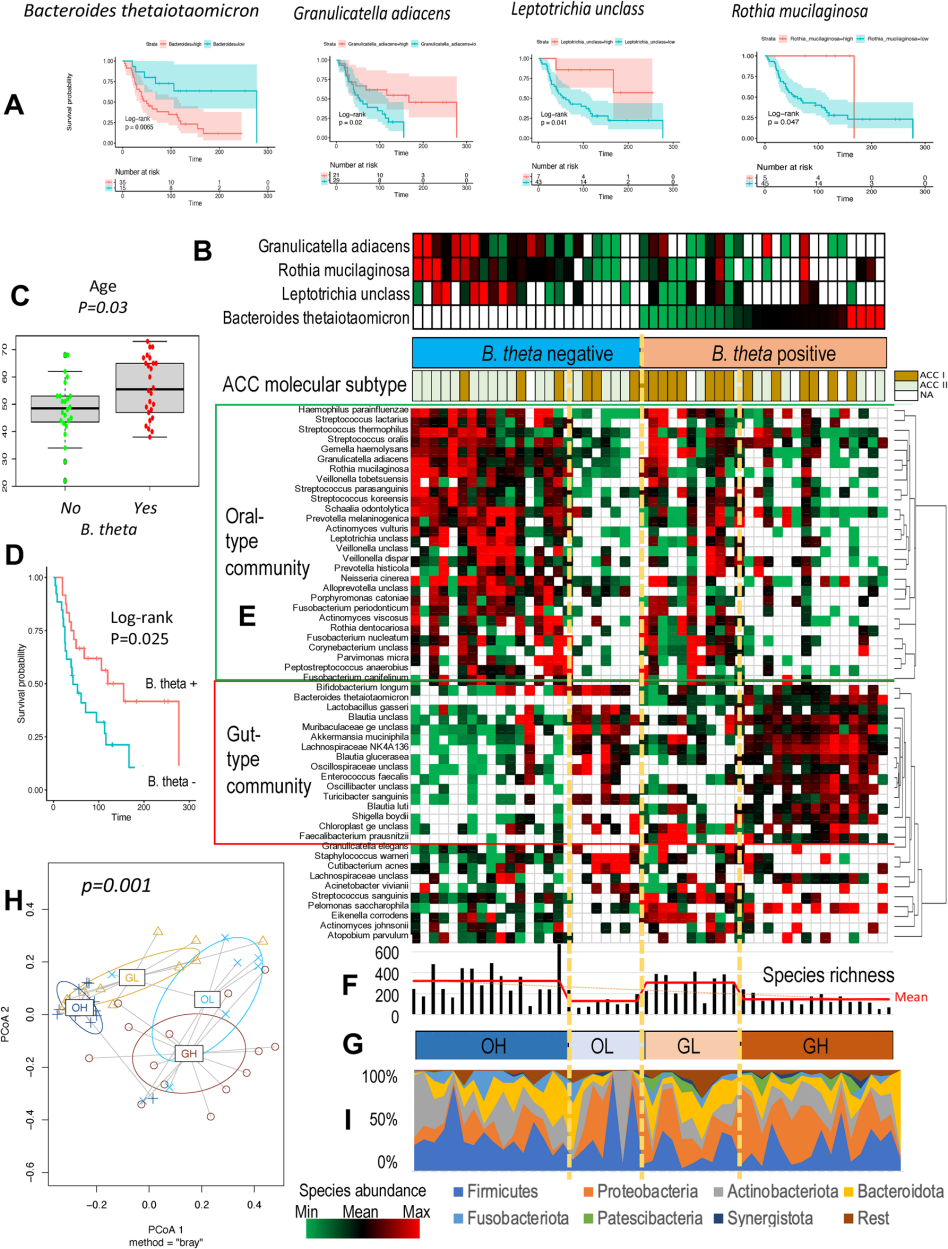
Grouping of ACC intratumoral microbiomes according to bacterial community type and abundance. (**A**) Bacterial species significantly associated with overall survival. (**B**) Grouping and ordering of ACCs according to the abundances of species associated with survival. The studied ACCs were subdivided into 2 groups: *B. theta*-positive and -negative. The former group was ordered by the sum of 3 OS-positive species abundances, from max to min, and the latter by *B. theta* abundance, from min to max. The *B. theta*-positive group of ACC tumors was dominated by the molecular subtype ACC-II, while the ACC-I subtype was significantly more common in *B. theta*-negative ACCs. (**C**) Association of *B. theta−* and *B.theta* + tumors with the age of the ACC patient. (**D**) Association of *B. theta−* and *B.theta* + tumors with ACC patient survival. (**E**) Supervised hierarchical clustering of the 54 most common spp. identified in ACCs reveals oral- and gut-type bacterial communities associated with *B. theta-*negative and -positive tumor groups, respectively. The oral-type bacterial community is enriched with common oral species, while the gut-type community is enriched with species that are common in the human gut. (**F**,**G**) Grouping of ACC intratumoral microbiomes according to oral and gut community abundances and associations of the groups according to richness; OH: high abundance of oral species, OL: low abundance of oral species, GH: high abundance of gut species, GL: low abundance of gut species. The OH and GL microbiomes had significantly greater richness than did the OL and GL microbiomes. (**H**) Permutational multivariate analysis of variance confirmed significant differences in the microbial composition of the proposed intratumoral microbiome groups (OH, OL, GH, and GL). (**I**) Significant association of the bacterial community taxonomic structure at the phylum level with the proposed grouping of the intratumoral microbiome in the ACC. *Firmicutes*, *Actinobacteriota*, and *Fusobacteriota* were more abundant in the *B. theta-*negative group, while the phyla *Proteobacteria*, *Patescibacteria,* and *Cyanobacteria* were more abundant in *B. theta* + tumors.

Among the topmost common and abundant species analyzed by the Cox proportional hazards model, only 1 OTU was significantly (P value = 8E−05) negatively associated with survival after adjustment of the log-rank p value for multiple comparisons (Fig. 2A). The OTU was classified taxonomically with 100% identity as a well-known commensal resident of the human gut^51^ *Bacteroides thetaiotaomicron* (*B. theta*). Importantly, the abundance of *B. theta* predicted survival probability independently of the ACC subtype, and the combination of *B. theta* abundance and ACC subtype increased the significance of the model from P = 2E−4 to P = 3E−5 according to the likelihood ratio test (Supplementary Table S3). Only 3 OTUs (Fig. 2A, Supplementary Table S2), taxonomically classified as typical inhabitants of the oral cavity (*Granulicatella adiacens*, *Rothia mucilaginosa*, and an unclassified species of *Leptotrichia*), had a significant positive association with survival (likelihood ratio test P, not adjusted; 0.03, 0.05, and 0.05, respectively). The abundances of the OTUs were significantly positively correlated with each other across the samples (rho ranged from 0.36 to 0.70), suggesting that the oral species can be a part of the same community. Each of the species had a significant negative correlation with *B.theta* abundance (rho ranged from − 0.27 to − 0.49), revealing their potential negative impact on the outgrowth of *B. theta*.

To explore the associations of the species with other topmost common colonizers of the intratumoral microbiome, we grouped tumors and species in terms of *B. theta* and oral species abundances (Fig. 2B,C). We divided the tumors into *B.theta* + (positive) and *B. theta−* (negative) groups and then ordered the *B. theta* + tumors by *B. theta* abundance from the maximum to 0 (from right to left); and *B. theta*-negative samples were ordered by the sum of the 3 oral species abundances from min to max (Fig. 2B). Patients with *B. theta− *tumors were younger (Fig. 2C) and had better outcomes (Fig. 2D), and their tumors were more likely to be of the ACC-II myoepithelial molecular subtype (Fisher’s test P = 0.01). No associations were found between the *B. theta−* or *B. theta* + phenotype and primary tumor site (Pearson's chi-square test P values of 0.23 and 0.37, respectively).

Supervised hierarchical clustering was used to group the topmost common species according to similar abundance profiles across the ordered tumors (Fig. 2E). Similar profiles of a set of species across different conditions would suggest their existence as a community. The clustering revealed 2 major types of bacterial communities, referred to as oral type (O-type) and gut type (G-type). The O-type communities were dominated by species that are typical for the oral cavity, including *Granulicatella adiacens*, *Rothia mucilaginosa*, and *Leptotrichia unclassified*. The G-type communities were dominated by known common gut bacteria, including *B. theta* and *Akkermansia muciniphila, which are* well-known mucus layer degraders in the gut*;* putative species of *Blautia* and *Lactobacillus gasserai;* and other bacteria that are not typical for humans as hosts.

Because the abundance of the O-type community varied in *B. theta−* tumors and decreased in tumors with low richness (Fig. 2F), we further subdivided the tumors into 2 subgroups (Fig. 2G): the Oral High (OH) group, which had a high abundance of oral species and high species richness in the intratumoral microbiome; and the Oral Low (OL) group, which had a low abundance of oral species and low species richness. In *B. theta* + tumors, the abundance of the G-type community also varied, but the abundance of gut species increased when the species richness was low (Fig. 2F). Therefore, the tumors were also subdivided into 2 subgroups: the gut high (GH) subgroup, which had a high abundance of gut species and low species richness in their intratumoral microbiomes, and the gut low (GL) subgroup, which had a low abundance of gut species and high species richness. The 4 identified subtypes of ACC intratumoral microbiomes, *B. theta−* OH and OL and *B. theta* + GH and GL, exhibited significant associations (nonparametric MANOVA P = 1e−04 by and PERMANOVA P = 0.001) with the taxonomic composition of the bacterial community (Fig. 2H,I). Species of *Firmicutes*, *Actinobacteriota*, and *Fusobacteriota* were more abundant in the *B. theta-*negative group (t test P = 0.06, P = 0.03, P = 0.10, respectively), while species of *Proteobacteriota* were significantly enriched in *B. theta* + tumors (t test P = 0.02), especially in samples characterized by low species richness (GH group), compared with the OH group (P = 0.002). Interestingly, *Patescibacteria* and *Cyanobacteria*, which are not common inhabitants of the oral cavity, had increased abundances in *B. theta* + tumors (t test P = 0.005 and P = 0.06, respectively).

**Figure 3:**
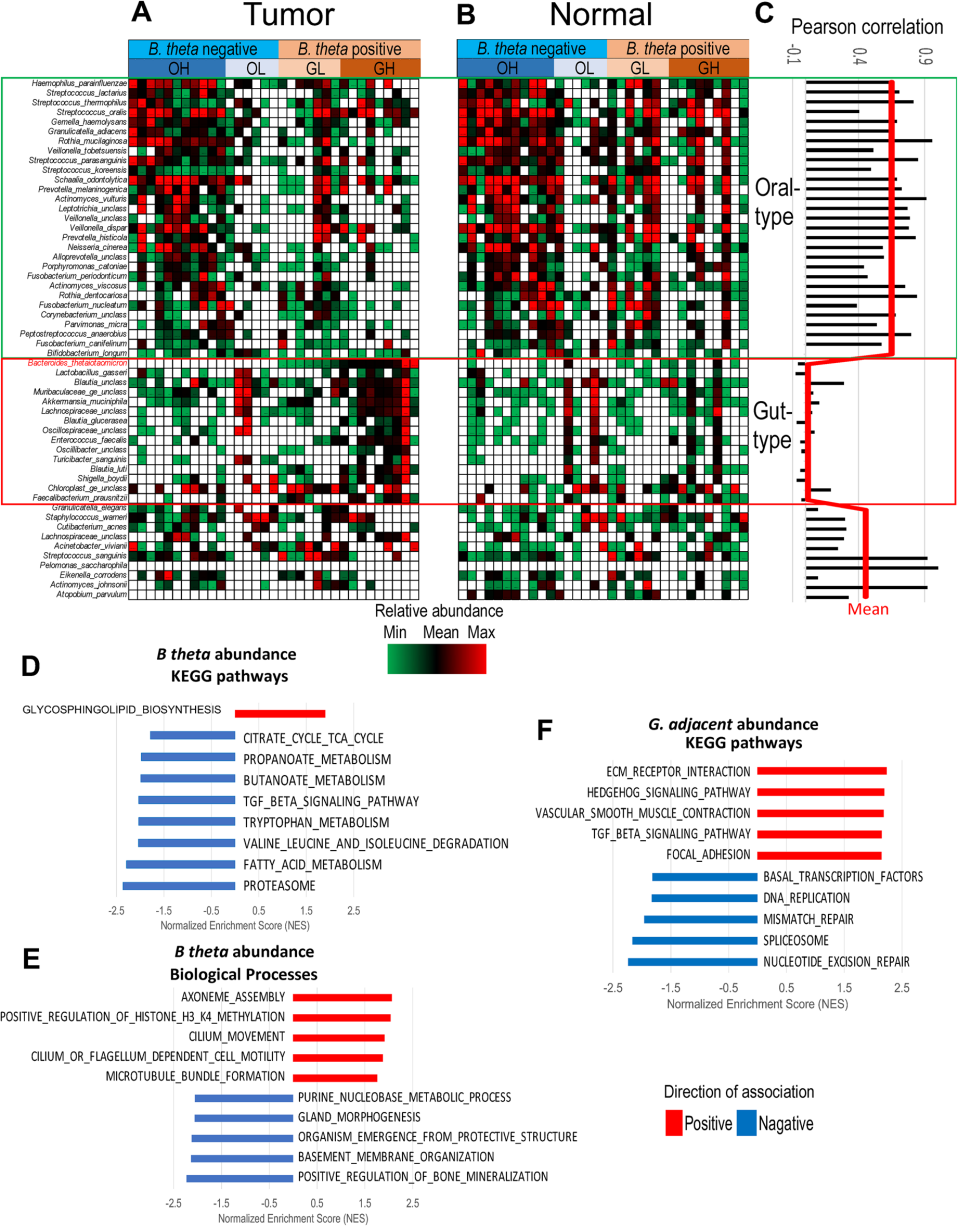
Distinct characteristics of the gut- and oral-type communities in ACC tumors. (**A**,**B**) Heatmaps of the most common species in 33 pairs of ACC (**A**) and adjacent normal (**B**) samples. The species and samples in both heatmaps are shown in the same order as in Fig. 2C. Species of the gut-type community are abundant in ACC tumors but are weakly abundant or absent in their adjacent normal counterparts. (**C**) Pairwise Person correlation analysis showing a significant association between tumor and adjacent normal species in the oral-type community and no association for species in the gut-type community. (**D**,**E**) KEGG pathway and biological process enrichment in ACC cells significantly associated with the abundance of *B. theta*. (**F**) KEGG pathways significantly associated with the abundance of *G. adiacens* in ACC tumors.

A significant positive correlation of species richness between paired tumor tissue and normal tissue in the present study (Supplementary Fig. S3) suggested that the dominance of certain oral or gut-associated species within the tumor might have occurred because bacteria from normal tissue colonized the developing tumor of the patient. Therefore, we explored the correlations of species abundances between tumor and normal tissues (Fig. 2E) in 33 paired samples to validate this hypothesis. Heatmaps of tumor and normal tissue paired with the same order of samples and OTUs as in Fig. 2E are presented in Fig. 3A,B, respectively. Correlation analysis for each species across 33 samples is provided in Fig. 3C. The results support the notion that tumors colonize oral species from normal tissue because their abundance in tumors significantly positively correlates with their abundance in normal tissue (mean R = 0.65, P < 0.02). The abundances of species composing the gut-type community in tumors were not correlated with those in normal tissues (mean R = 0.02; P > 0.1), suggesting de novo colonization of the tumor by non-oral, mainly gut-associated species.

### Biological processes correlated with the abundances of *B. theta* and *G. adiacens* in ACC tumors

To identify biological processes correlated with the abundances of *B. theta* and *G. adiacen*s in ACC tumors, we interrogated our previous RNA sequencing^4^ data for 44 ACC tumors via GSEA. The analysis (Fig. 3D) revealed only one KEGG pathway, biosynthesis of glycosphingolipids, that was significantly positively correlated with the *B. theta* abundance in ACC tumors (FDR = 0.03). Glycosphingolipids (GSLs) are known components of the cell membrane. They are composed of ceramide backbones and glycans that remain outside of the membrane and participate in cell adhesion, cell‒cell interactions, and migration^52–55^. The increased production of GSLs in tumors with abundant *B. theta* suggested that the availability of glycans for nutritional benefit might underlie the outgrowth of the bacterium. We also highlighted the activities of related processes, including assembly of the axoneme, cilium movement, and cilium-dependent cell motility (Fig. 3E)^56^, among the top biological processes positively correlated with *B. theta*. In contrast, activities associated with the normal structure and organization of the salivary gland, such as gland morphogenesis and basement membrane organization, were negatively correlated with *B. theta* abundance (Fig. 3E).

Several molecular pathways known to be significantly more active in the ACC-II molecular subtype^4^, including extracellular matrix receptor interactions, focal adhesion, Hedgehog, and TGF-beta signaling pathways, were positively correlated with *G. adiacens* abundance (Fig. 3F). Likewise, processes associated with cell proliferation, a known signature of the ACC-I molecular subtype, were negatively correlated with *G. adiacens* abundance.

### Comparison of intratumoral microbiomes in ACC patients with oral and head and neck cancers

**Figure 4:**
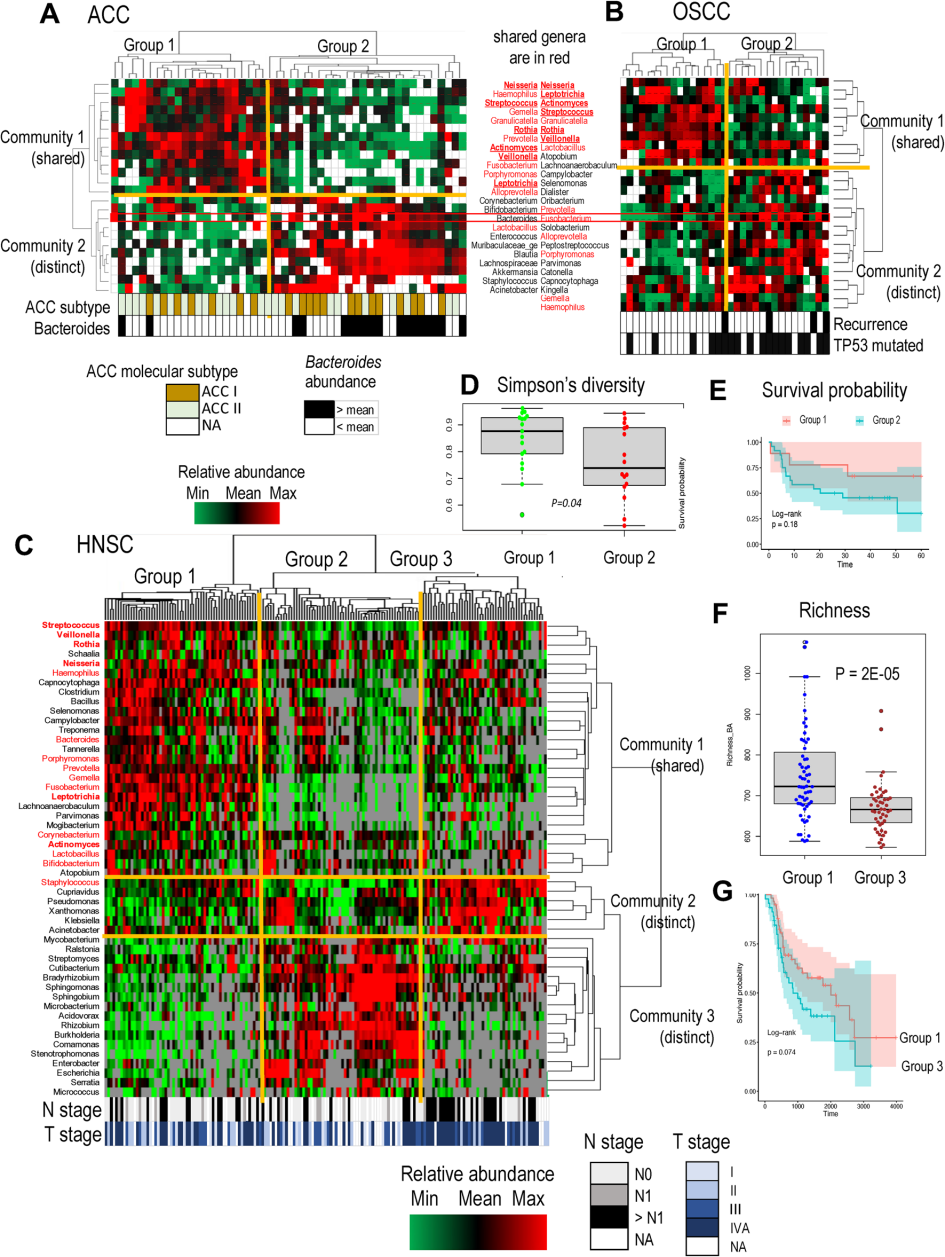
Comparative analysis of the intratumoral microbiomes of ACC patients with those of OSCC and HNSC patients. (**A**–**C**) Unsupervised hierarchical clustering of tumors in the ACC (**A**), OSCC (**B**), and HNSC (**C**) cohorts identified 2 bacterial communities (rows) in each cohort: Community 1 populated by shared oral *genera* (names are colored in red) and Community 2 populated by *genera* that are mainly specific for each cohort (names are colored in black). The names of the genera identified in all three cohorts are given in red bold. The columns of each heatmap denote tumors that are clustered together into major tumor groups (top of each heatmap) with similar abundance profiles of the intratumoral communities. The bars at the bottom of each heatmap show characteristics associated with the tumor groups. In the ACC (**A**), clustering revealed 2 tumor groups that recapitulated the supervised clustering (Fig. 2B,E) based on intratumoral *B. theta* abundance. Group 1 ACC tumors had many healthy oral genera but less abundant *Bacteroides* (t-test P = 0.0006) and other gut-associated *Genera*. The aggressive ACC I molecular subtype was also less common in patients in the other groups (Fisher’s test P = 0.14). In the OSCC cohort (**B**), Group 1 tumors had less abundant *Fusobacterium* (t test P = 0.0001), a significantly reduced frequency of TP53 mutations (Fisher’s test P = 0.037) and disease recurrence (Fisher’s test P = 0.044). *Bacteroides* and *Fusobacterium* are labeled by red rectangles in ACC and OSCC, respectively, as potential biomarkers of a more aggressive phenotype. In HNSC (**C**), clustering revealed 3 tumor groups (Group 1, Group 2, and Group 3). Group 1 HNSC tumors were less likely than Group 3 HNSC tumors to be at an advanced stage (Fisher’s test P = 0.01) or to spread into the lymph nodes (Fisher’s test P = 0.05). No difference was found between Group 1 and Group 2 tumors. (**D**,**E**) Community 1 in OSCC patients was associated with a more diverse microbiome (**D**) and better overall patient survival (**E**). (**F**,**G**) Community 1 in HNSC was also associated with a more diverse microbiome (**F**) and better overall patient survival (**G**).

The bacterial communities identified in ACC may resemble those found in oral squamous cell carcinoma (OSCC) and head and neck squamous cell carcinoma (HNSC), as all three cancers originate in or near the oral cavity. To identify the bacterial genera that cancers may share with ACC, we examined previously published datasets of the intratumoral microbiomes of these cancers. We selected the most common genera from each of the three OTU tables and clustered each table using unsupervised hierarchical clustering with the same parameters (Fig. 4A–C). Clustering identified 2 communities of bacterial *Genera* (Community 1 and Community 2) associated with each cancer. Many *Genera* were shared between ACC and OSCC (36%) and between ACC and HNSC (34%) and clustered in Community 1, which is also referred to as shared. Importantly, 6 *Genera* of the healthy oral microbiome, *Neisseria, Leptotrichia, Actinomyces, Streptococcus, Rothia, and Veillonella,* were shared among all three cancers and clustered close to each other in shared Community 1. Like that in ACC, the bacterial community in OSCC and HNSC tumors was enriched and characterized by a more diverse intratumoral microbiome (Fig. 4D,F, respectively), a less aggressive phenotype (Fig. 4B,C, respectively), and better overall patient survival (Fig. 4E,G, respectively). Thus, the shared oral genera may be considered biomarkers of healthier tumor microbiomes in all three cancers. In contrast, Community 2 was composed of distinct bacteria from each of the three cancers and was associated with a more aggressive phenotype and worse patient survival.

## Discussion

**Figure 5:**
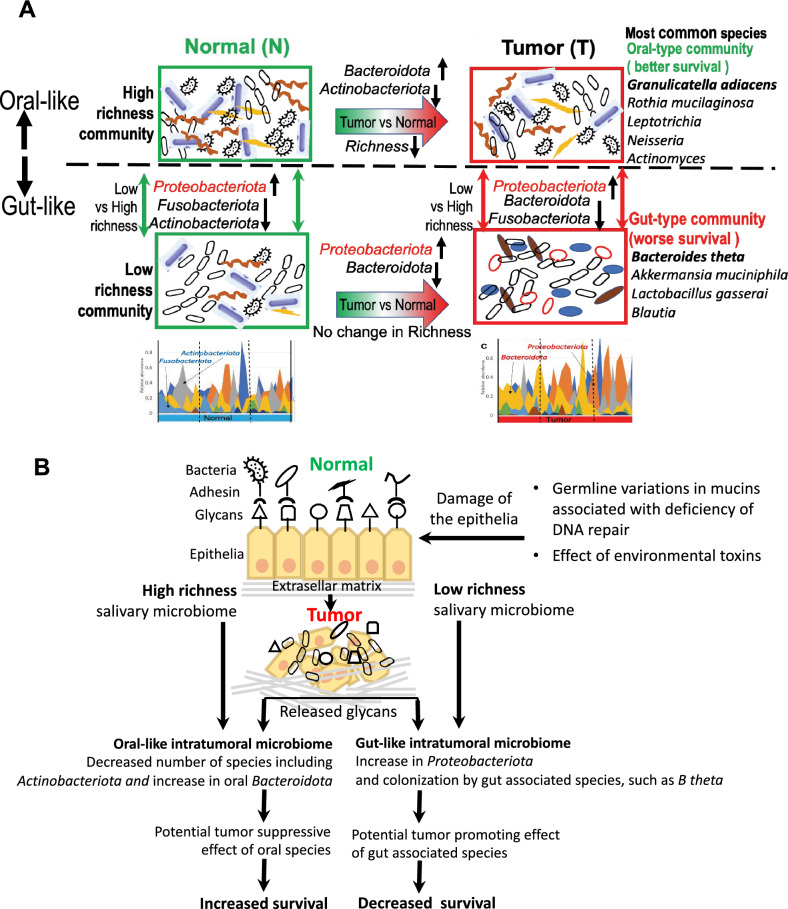
Graphical summary of the results. (**A**) Distinct taxonomic structure of the intratumoral microbiome in ACC patients with high and low species richness and association with survival. A rich microbial community in normal tissue is linked to an oral-type intratumoral community dominated by oral species with significantly increased abundances of *Bacteroidota* and decreased abundances of *Actinobacteroidota*. A microbial community with low richness is linked to the gut type intratumoral community populated by many species involved in mucus layer degradation. (**B**) Hypothetical model linking ACC development and progression to distinct changes in the taxonomic composition of the intratumoral microbiome and feedback effects. Sustained damage to salivary epithelia during the course of ACC development leads to the release of salivary glycans and the outgrowth of glycan-degrading bacterial taxa. In the low-richness intratumoral microbiome, which is not resilient, the outgrowing taxa are represented by non-oral gut-associated bacteria and *Proteobacteria* that promote tumor progression. In the high-richness intratumoral microbiome, which is more resilient, the taxa are represented by oral *Bacteroidota*. Although most other oral species are decreased, colonization by gut-associated glycan degraders is suppressed by oral microbes.

The study revealed two major microbial subtypes, oral-like and gut-like, in the enclosed salivary ACC (Fig. 5A). Tumors of the subtypes are characterized by different microbial communities (oral or gut type), different tumor phenotypes (less or more aggressive), associations with different molecular subtypes of ACC (ACC-II or ACC-I), and better or worse probabilities of overall survival. Patients with an increased number of bacterial species (high richness) in normal tissue have oral-type normal and tumor microbiomes that are similar in taxonomic composition and populated by many shared oral taxa, such as *Granulicatella*, *Rothia*, and *Leptotrichia*; these taxa are positively associated with patient survival. Because the submandibular and minor salivary glands were the main sites of ACC in the cohort (62% of samples), it is expected that the bacterial communities of the glands would be shared with those of the oral cavity. According to a published study of bacterial microbiomes from healthy humans^57^, oral sites had high relative abundances of *Streptococcus, Veillonella*, *Prevotella*, *Neisseria*, *Actinomyces*, *Leptotrichia, Rothia*, and *Porphyromonas.* The same genera were found to be abundant in the oral type of microbial community in ACC tumor and in normal tissues (Fig. 4B–D). Furthermore, a comparative analysis of the intratumoral microbiomes of ACC patients in this study with those of OSCC and HNSC patients revealed that 6 of these oral genera were associated with a more diverse intratumoral community, better survival, and a less aggressive tumor phenotype in all three cancers. These findings suggest the co-occurrence and interaction of these oral genera as part of a bacterial community and highlight their potential role in tumor suppression not only in the ACC but also in other cancers associated with the oral cavity. The positive effect of the oral community on survival can be due to the favorable tumor microenvironment supporting the diversity, functional redundancy, resilience and stability of not only the bacterial *Genera* but also the host immune cells in the microenvironment^46,58–60^. In addition to these tumor suppressive effects, there are likely additional specific molecular mechanisms involved. In our study, the most significant positive effect on survival in the ACC was found for *Granulicatella adiacens*, a common inhabitant of normal oral microbiota. This effect can be explained by the nutritional requirements of this fastidious bacterium, which requires vitamin B_6_ (pyridoxine) for growth^61^. Because pyridoxine must be supplied from the environment, increased intake of this vitamin by growing *G. adiacens* may reduce the availability of *B*_*6*_ for proliferating ACC cells^62^. However, further research is needed to provide evidence of the involvement of *G. adiacens* in ACC pathogenesis and to explore the specific underlying molecular mechanisms involved.

The gut-like microbial subtype was found in ACC tumors with reduced species richness in normal salivary tissue. This subtype is associated with dramatic changes in the taxonomic structure of intratumoral microbiomes, suggesting an outgrowth of taxa that are not typical of the oral cavity. Multiple new colonizers of these communities are common inhabitants of the human gut, including *B. thetaiotaomicron*, which has a significant negative association with patient survival. Although low species richness was not linked to a distinct taxonomic structure of the intratumoral microbiome in any cancer, the abundant gut species and *Proteobacteria* observed in ACC tumors with low richness are not surprising and can be explained by the low resistance of such bacterial communities to colonization by other species, including pathogens^58,63^. Indeed, *Proteobacteria* was significantly expanded in low-richness ACCs. The *Phylum* is a known microbial signature of human diseases and impaired epithelial tissue^64–66^. It is also strongly associated with metastasis and poor survival in cancer patients^67^. Other uncommon ACC colonizers, including gut microbes from the *Genera Bacteroides, Akkermansia, Blautia, Enterococcus, Faecalibacterium, Fusobacterium, and Bifidobacterium*^57,68^, can also be linked to metabolic and structural changes in tumor tissues^69^. Several studies have reported not only the presence but also the involvement of oral microbes in the pathogenesis of gastrointestinal inflammatory diseases^45,70^ and colon cancer^71^. Disease-associated metabolic shifts in the gut may drive the colonization of the gut by oral *Veillonella parvula* or *Fusobacterium nucleatum*. In our study, we *observed* a reverse oral-gut microbiome interaction, in which low-richness ACC tumors were colonized by gut microbes. It is also possible that other occasional nonhuman inhabitants, such as the plant-associated *Bradyrhizobium centrosematis*^72^, can colonize tumors with low richness. Bacteria are also common pollutants in drinking water^49^ and have been reported as NGS contaminants^73,74^. However, in our study, *B. centrosematis* was found to be differentially abundant only in tumors with a low number of species but not in normal tissue or in tumors with a rich microbiome. Therefore, expansion of the bacterium in tumors may be relevant to ACC pathogenesis. Additional experimental studies are needed to confirm these findings.

Notably, several intratumoral bacteria, including *Akkermansia, Bacteroides, Blautia, Bifidobacterium,* and *Enterococcus*, found in low*-*richness ACCs are not only known human gut colonizers but can also utilize mucus glycans for growth^75^. In ACCs with high richness, oral species of *Bacteroidota,* known for their consumption of mucus glycans, also exhibited significantly greater abundances than did matched normal tissue. We propose, therefore, that the release of glycans by developing tumors may lead to the expansion of glycan degraders (Fig. 5B). Glycan liberation in ACC can result from sustained damage to the salivary epithelium due to DNA repair pathway deficiencies and an increased frequency of rare germline mutations in mucins that defend epithelial tissue against toxins^76^. Consistent with this hypothesis, GSEA of tumor RNA revealed that the biosynthesis of cell membrane components composed of glycans was significantly positively correlated with *B. theta* abundances and that activities associated with the normal organization of the salivary gland were negatively correlated (Fig. 3D,E). Expansion of glycan-degrading bacteria may promote the development and progression of ACC through positive feedback effects on tumor growth. Degradation of salivary glycans damages the mucus layer that protects epithelial cells from the environment and helps to maintain healthy tissue in the salivary gland^22,77^. The degradation may also produce byproducts, such as ornithine and ceramides, which have tumor-promoting effects^78^. In addition, the release of monosaccharides as byproducts of mucus degradation^79^ can lead to the growth and reprogramming of tumor cells. However, further studies are necessary to determine the tumor-promoting effects of *B.theta* and other mucus degraders and to reveal the underlying molecular mechanisms involved.

## Conclusions

Two microbial subtypes dominated by oral versus gut bacterial species were identified in the ACC. The oral-like subtype is associated with a less aggressive tumor phenotype, an ACC-II molecular subtype, and a better probability of patient survival. The dominant *Genera* in the subtypes were shared with the oral community found in healthy individuals and with the intratumoral microbiomes of OSCC and HNSC patients. In both cancer types, intratumoral communalities with shared oral species were associated with a more diverse microbiome, less aggressive tumor characteristics, and better survival. The gut-like ACC microbial subtype is characterized by an increased abundance of known mucus layer degraders dominated by gut species in low-richness microbiomes and by oral *Bacteroidota* in high-richness microbiomes. The most significant negative association with survival in the ACC was observed for *B. theta*, likely because of damage and reprogramming of salivary epithelia during ACC progression, resulting in the release of glycans consumed by the organism.

### Supplementary Information

Supplementary Figures.

Supplementary Tables.

## Data Availability

The 16S rRNA gene sequencing data were deposited into the NCBI Sequence Read Archive (http://www.ncbi.nlm.nih.gov/sra) under the BioProject accession number PRJNA1049986.
